# Role of the Steroid Sulfate Uptake Transporter Soat (*Slc10a6*) in Adipose Tissue and 3T3-L1 Adipocytes

**DOI:** 10.3389/fmolb.2022.863912

**Published:** 2022-04-28

**Authors:** Emre Karakus, Andreas Schmid, Silke Leiting, Bärbel Fühler, Andreas Schäffler, Thilo Jakob, Joachim Geyer

**Affiliations:** ^1^ Institute of Pharmacology and Toxicology, Faculty of Veterinary Medicine, Justus Liebig University, Giessen, Germany; ^2^ Department of Internal Medicine III, Giessen University Hospital, Justus Liebig University, Giessen, Germany; ^3^ Department of Dermatology and Allergology, Giessen University Hospital, Justus Liebig University, Giessen, Germany

**Keywords:** SOAT, Slc10a6, DHEAS, transport, knockout mouse, 3T3-L1, adipocytes, adipogenesis

## Abstract

In addition to the endocrine and paracrine systems, peripheral tissues such as gonads, skin, and adipose tissue are involved in the intracrine mechanisms responsible for the formation of sex steroids *via* the transformation of dehydroepiandrosterone and dehydroepiandrosterone sulfate (DHEA/DHEAS) into potent androgenic and estrogenic hormones. Numerous studies have examined the relationship between overweight, central obesity, and plasma levels of DHEA and DHEAS. The sodium-dependent organic anion transporter Soat (*Slc10a6*) is a plasma membrane uptake transporter for sulfated steroids. Significantly increased expression of *Slc10a6* mRNA has been previously described in organs and tissues of lipopolysaccharide (LPS)-treated mice, including white adipose tissue. These findings suggest that Soat plays a role in the supply of steroids in peripheral target tissues. The present study aimed to investigate the expression of Soat in adipocytes and its role in adipogenesis. Soat expression was analyzed in mouse white intra-abdominal (WAT), subcutaneous (SAT), and brown (BAT) adipose tissue samples and in murine 3T3-L1 adipocytes. In addition, adipose tissue mass and size of the adipocytes were analyzed in wild-type and *Slc10a6* ^*−/−*^ knockout mice. Soat expression was detected in mouse WAT, SAT, and BAT using immunofluorescence. The expression of *Slc10a6* mRNA was significantly higher in 3T3-L1 adipocytes than that of preadipocytes and was significantly upregulated by exposure to lipopolysaccharide (LPS). *Slc10a6* mRNA levels were also upregulated in the adipose tissue of LPS-treated mice. In *Slc10a6* ^*−/−*^ knockout mice, adipocytes increased in size in the WAT and SAT of female mice and in the BAT of male mice, suggesting adipocyte hypertrophy. The serum levels of adiponectin, resistin, and leptin were comparable in wild-type and *Slc10a6* ^*−/−*^ knockout mice. The treatment of 3T3-L1 adipocytes with DHEA significantly reduced lipid accumulation, while DHEAS did not have a significant effect. However, following LPS-induced Soat upregulation, DHEAS also significantly inhibited lipid accumulation in adipocytes. In conclusion, Soat-mediated import of DHEAS and other sulfated steroids could contribute to the complex pathways of sex steroid intracrinology in adipose tissues. Although in cell cultures the Soat-mediated uptake of DHEAS appears to reduce lipid accumulation, in *Slc10a6* ^*−/−*^ knockout mice, the Soat deletion induced adipocyte hyperplasia through hitherto unknown mechanisms.

## Introduction

Steroid hormones play a vital role in the regulation of many physiological processes, including mineral and glucose homeostasis and sexual differentiation. In addition to gonadal steroid secretion, humans and other species have evolved intracrine mechanisms of androgenic and estrogenic steroid formation in peripheral tissues such as skin and adipose tissue (Labrie et al., 1991, Labrie et al., 2005; Dhatariya and Nair, 2003; Nawata et al., 2004). The sulfated form of dehydroepiandrosterone (DHEA), namely, DHEAS is the most abundant circulating steroid hormone in humans and is secreted exclusively by adrenal glands. The precursor hormone DHEAS acts as a large reservoir for the synthesis of potent androgens and estrogens in peripheral tissues (Labrie et al., 1991, Labrie et al., 2005), which naturally possess the enzymatic machinery to transform DHEAS into active sex steroids (Killinger et al., 1995; Labrie et al., 2001). The plasma levels of DHEA and DHEAS decrease with aging in both men and women (Labrie et al., 2003). Furthermore, a reduction in DHEA and/or DHEAS levels has been linked to age-related conditions such as cardiovascular disease (Wu et al., 2017), prostate and breast tumors (Zumoff et al., 1981; Schwartz et al., 1986; Stahl et al., 1992), immune deficiency (Casson et al., 1993), poor mental health (Haren et al., 2007; Wong et al., 2011), and insulin resistance. Low serum levels of DHEAS have also been associated with rheumatoid arthritis (Deighton et al., 1992) and with certain features of obesity, such as high body mass index (Tchernof et al., 1995), central fat accumulation (De Pergola et al., 1991, De Pergola et al., 1996), and the increase in visceral fat in men and waist-to-hip ratio in women (Hernández-Morante et al., 2008; Sayın et al., 2020). These correlations remained significant after adjustment for age. Interestingly, *in vitro* studies on adipocytes have revealed the stimulatory effect of DHEAS on lipolysis, suggesting that elevated levels of DHEAS negatively influence lipid accumulation (Hernández-Morante et al., 2008).

Clinical trials have also demonstrated that the administration of DHEA has beneficial effects on several obesity-associated pathologies, including improving insulin resistance and glucose metabolism in type 2 diabetic patients, decreasing body fat mass, and even decreasing abdominal visceral and subcutaneous fat in elderly men and women (Nestler et al., 1988; Yen et al., 1995; Morales et al., 1998; Yamaguchi et al., 1998; Villareal and Holloszy, 2004; Weiss et al., 2011). Moreover, in rat and mouse studies, DHEA or DHEAS treatment reduced body weight gain, lipid accumulation, number of fat pads, body fat percentage, and adipocyte levels (Tagliaferro et al., 1986; Muller and Cleary, 1985; Lea-Currie et al., 1997a, Lea-Currie et al., 1997b). In addition, DHEAS was found to reduce adipocyte hyperplasia in rats on a high-fat diet (Lea-Currie et al., 1997b). Furthermore, *in vitro* DHEA decreased the proliferation of preadipocytes and reduced their differentiation into mature adipocytes (Gordon et al., 1986; Shantz et al., 1989; Lea-Currie et al., 1998).

DHEAS is negatively charged at physiological concentrations and, therefore, cannot enter cells by diffusion, which is a key point that has not been sufficiently considered in these studies. Specific uptake transporters are required to transport DHEAS from the extracellular to intracellular compartment. The steroid sulfate uptake transporters belonging to the solute carrier families of organic anion transporting polypeptides (OATPs) and organic anion transporters (OATs) all represent multispecific carriers (Roth et al., 2012). However, the sodium-dependent organic anion transporter SOAT (gene symbol *SLC10A6* in humans and *Slc10a6* in animals) that belongs to the solute carrier family SLC10 is a specific uptake transporter for sulfated steroid hormones. DHEAS, 16α-hydroxy-DHEAS, pregnenolone sulfate, 17α-hydroxypregnenolone sulfate, androstenediol-3-sulfate, epiandrosterone-3β-sulfate, androsterone-3α-sulfate, testosterone-17β-sulfate, epitestosterone-17α-sulfate, 5α-dihydrotestosterone sulfate, estrone-3-sulfate, and 17β-estradiol-17-sulfate, representing the physiologically most relevant sulfated steroids, all have been identified as SOAT substrates (Geyer et al., 2004, Geyer et al., 2006, Geyer et al., 2007; Fietz et al., 2013; Schweigmann et al., 2014; Grosser et al., 2018). After their cellular uptake through SOAT/Soat transporters and intracellular desulfation by steroid sulfatase (STS), these sulfated steroids contribute to the steroid regulation of peripheral tissues such as adipose tissue or skin in an intracrine manner (Reed et al., 2005; Labrie et al., 1991, Labrie et al., 1998, Labrie et al., 2005). *SLC10A6* mRNA is highly expressed in the testes, pancreas, placenta, breast, lung, heart, skin, vagina, and kidney in humans (Geyer et al., 2007; Fietz et al., 2013). According to data from the Human Protein Atlas (2022), SOAT protein is present in the breast, bronchus, cervix, esophagus, nasopharynx, oral mucosa, prostate, skin, stomach, tonsil, and vagina, all at medium score expression levels. In addition, SOAT protein is highly expressed in various breast pathologies, including ductal hyperplasia, intraductal papilloma, and intraductal carcinoma (Karakus et al., 2018). In mice, the expression of *Slc10a6* mRNA has been detected in the lungs, testes, heart, bladder, pancreas, gall bladder, and skin. Furthermore, by immunohistochemistry, Soat protein was localized in bronchial epithelial cells of the lung, in primary and secondary spermatocytes, in the round spermatids within the seminiferous tubules of the testis, in the epidermis of the skin, and in the urinary epithelium of the bladder (Grosser et al., 2013). In a recent study, *Slc10a6* mRNA was among the most strongly induced transcripts in mice exposed to lipopolysaccharide (LPS), suggesting that *Slc10a6* represents an inflammation-responsive gene (Kosters et al., 2016).

Based on these previous findings, the present study aimed to extend our knowledge of the role of Soat in adipogenesis by investigating the mRNA and protein expression pattern of *Slc10a6*/Soat in adipocytes. The experiments were performed *in vivo* in *Slc10a6* ^*−/−*^ knockout and wild-type mice and in LPS-treated mice and *in vitro* using the 3T3-L1 adipocyte cell line.

## Materials and Methods

### Materials

All chemicals, unless otherwise stated, were obtained from Sigma-Aldrich (Taufkirchen, Germany).

### Cell Culture

Murine 3T3-L1 preadipocytes were cultured at 37 °C and 5% CO_2_ in DMEM (Dulbecco’s modified Eagle medium, Biochrom AG, Berlin, Germany) supplemented with 10% newborn calf serum (NCS; Sigma-Aldrich) and 1% penicillin/streptomycin (PAN-Biotech, Aidenbach, Germany). At confluence, the cells were differentiated into mature adipocytes by culturing in DMEM/F12/glutamate medium (Lonza, Basel, Switzerland) supplemented with 20 μM 3-Isobutylmethylxanthine (Serva, Heidelberg, Germany), 1 μM corticosterone, 100 nM insulin, 200 μM ascorbate, 2 μg/ml apo-transferrin, 5% fetal calf serum (FCS, PAN-Biotech), 1 μM biotin, 17 μM pantothenate, 1% penicillin/streptomycin, and 300 μg/ml Pedersen fetuin (MP Biomedicals, Illkirch, France) (Zaitsu and Serrero, 1990; Bachmeier and Löffler, 1994) for 9 days using a slightly modified protocol as reported previously (Hochberg et al., 2021). The cell cultures were supplemented with adipogenesis-inducing medium (AIM) from day 0 to day 8 of differentiation, when the cell culture medium was switched to serum-free conditions (with increased insulin supplementation to a concentration of 1 μM). On day 9, the mature adipocytes were switched to serum-free DMEM/F12/glutamate-medium lacking insulin for 3–5 h. With the start of the simulation experiments, the cells were supplied with a fresh serum-free medium. The cell phenotypes were established by light microscopy as having the appearance of extensive accumulation of lipid droplets. The mature adipocytes on day 9 of differentiation were used for stimulation experiments after overnight incubation under serum-free culture conditions.

### Real-Time PCR Analysis

The cells were stimulated with 10 ng/ml LPS (Sigma-Aldrich) for different periods of time. The cells were harvested and total mRNA was extracted using the Maxwell RSC simplyRNA Tissue Kit (Promega). Complementary DNA (cDNA) was synthesized from 1 μg total RNA using 8 μL of RT-mix SuperScript III Reverse Transcriptase (18080044, Invitrogen, Darmstadt, Germany). For quantitative analysis of the expression of the *Slc10a6*, *Slco1a1*, *Slco1a4*, and *Slco1b2* mRNA transcripts, gene-specific TaqMan Gene Expression Assays Mm00512730_m1, Mm01267415_m1, Mm00453136_m1, and Mm00451510_m1 (Thermo Fisher Scientific, Waltham, Massachusetts, United States) were used, respectively. The Mm99999915-g1 assay (Thermo Fisher Scientific) was used for the control amplification of glyceraldehyde-3-phosphate dehydrogenase (*Gapdh*). In the case of *Slco2b1* mRNA, real-time PCR was performed using the fluorescent dye SYBR Green PCR Master Mix (PowerUp SYBR Master Mix A25742, Thermo Fisher Scientific) and the following primers: *Slco2b1* forward 5′-AGT TTG AGC AGG GCT TCT ACC-3′ and *Slco2b1* reverse 5′-CTG TGA CAT AGG ACA AAG AAC TTG A-3’. The results were normalized to the mRNA expression of *Gapdh* (forward primer 5′-ACG GGA AGC TCA CTG GCA TG-3′ and reverse primer 5′-CCA CCA CCC TGT TGC TGT AG-3′). A standard curve analysis with these primer pairs revealed *r* ^2^ of 0.999 and PCR efficiency of 106.6% for *Slco2b1* and *r* ^2^ of 0.999 and PCR efficiency of 99.6% for *Gapdh*. The TaqMan expression assay efficiencies were as follows: *r* ^2^ of 0.998 and PCR efficiency of 97.2% for *Slc10a6*, *r* ^2^ of 0.994 and PCR efficiency of 96.5% for *Slco1a1*, *r* ^2^ of 0.996 and PCR efficiency of 103.6% for *Slco1a4*, *r* ^2^ of 0.991 and PCR efficiency of 98.7% for *Slco1b2*, and *r* ^2^ of 0.997 and PCR efficiency of 98.5% for *Gapdh*. Real-time amplification was performed on an Applied Biosystems 7300 Real-Time PCR System in 96-well optical plates using 5 µL cDNA, 1.25 µL TaqMan Gene Expression Assay, 12.5 µL TaqMan Universal PCR Master Mix, and 6.25 µL water in each 25 µL reaction. The plates were heated for 10 min at 95 °C, and then 40 cycles of 15 s at 95 °C and 60 s at 60 °C were applied. All data were expressed as fold changes using the 2^−ΔΔCt^ method with the calibrator indicated in the *Figure legends*. In the case of carrier expression profiling, ΔCt values are presented.

### Immunofluorescence

The cells were grown on poly-l-lysine–coated 8-well *μ*-slides (80826, IBIDI, Gräfelfing, Germany). After 48 h, the cells were incubated with or without 10 ng/ml LPS for 24 h and, afterward, were fixed with 2% paraformaldehyde (PFA) and blocked with blocking buffer containing 1% bovine serum albumin and 4% goat serum in phosphate-buffered saline (PBS, containing 137 mM NaCl, 2.7 mM KCl, 1.5 mM KH_2_PO_4_ (Roth), 7.3 mM Na_2_HPO_4_ (Roth), pH 7.4) for 30 min at room temperature. Then, 3T3-L1 cells were incubated at 4°C overnight with an antibody against murine Soat_329-344_ (1:500 dilution, Eurogentec, Seraing, Belgium), followed by labeling with the secondary antibody Alexa Fluor 488 goat anti-rabbit (1:800 dilution, A-11008, Invitrogen) and the nuclear marker Hoechst 33342 (1:5000, H1399, Invitrogen). Cell imaging was performed at room temperature on an inverted Leica DM5500 fluorescence microscope (Leica Microsystems, Wetzlar, Germany).

### Oil Red O Staining and the Triglyceride Assay

During adipocyte differentiation (days 0–9), the cells were treated with 1 µM DHEA, 100 nM testosterone, 10 µM DHEAS, 10 µM STX64, or 1 µM flutamide in the presence or absence of LPS (10 ng/ml) in 6-well plates. The cells were then washed with cold PBS and fixed with 4% formalin (v/v) for 30 min. After Oil Red O staining, the cells were photographed using a phase-contrast microscope (DMi1, Leica Microsystems) at ×10 magnification. For quantitative measurement of lipid accumulation, the stained cells were dried, Oil Red O was extracted in isopropanol, and absorbance was determined at 520 nm using a microplate reader.

### Sample Preparation From Mouse Tissues

Male C57BL/6 wild-type mice (age 8–12 weeks, bred on standard chow) received an intraperitoneal injection of 1 μg LPS per animal after overnight fasting. The control animals received intraperitoneal injections of the corresponding volume of H_2_O instead of LPS. The mice were euthanized for organ and tissue resection 2 h after injections. Epididymal (intra-abdominal) and subcutaneous adipose tissue specimens were resected and shock-frozen in liquid nitrogen for subsequent analysis of mRNA expression. Animal experiments were performed at the University of Regensburg, Germany, and all the animal studies were approved by the local government agency (approval no. 54-2532, 1.14/10). Furthermore, the organ samples were obtained from wild-type (WT) and *Slc10a6* ^*−/−*^ knockout mice after anesthesia with ketamine (120 mg/kg) and xylazine (16 mg/kg) and exsanguination by heart puncture. These experiments, including euthanasia and tissue preparations, were approved by the local regulatory authority (Regierungspräsidium Giessen) with the reference number V54-19 c 20 15 h 02 GI 20/23 Nr. A8/2013.

### Immunofluorescence Analysis on Tissue Sections

For histological analyses, the tissue samples from WT and *Slc10a6^−/−^* knockout mice were fixed for 10 min in 4% PFA and embedded in Tissue-Tek O.C.T. (Sakura Finetek Germany GmbH, Staufen, Germany). The fixed sections were permeabilized, blocked with 5% goat serum in PBS-TritonX-100 (0.1%), and incubated with the primary Soat antibody (mSoat_329-344_, 1:400 dilution, Eurogentec) overnight at 4°C. Subsequently, the tissue sections were incubated with the secondary antibody Alexa Fluor 488 goat anti-rabbit (1:800 dilution, A-11008, Invitrogen). The slides were counterstained with Hoechst 33342 and analyzed on a Leica DM5500 B microscope. The captured images were analyzed with Leica Fluorescence Workstation LAS AF 6000 software (Leica Microsystems).

### Measurement of Adipocyte Size

The adipose tissues from WT and *Slc10a6^−/−^* knockout mice were fixed in 10% formalin and processed for paraffin embedding. Paraffin tissue sections were cut into 3.5-µm slices and processed for hematoxylin and eosin (H&E) staining. For quantification of the size of the adipocytes, six tissue sections of each group were selected and photographed using the DMi1 microscope (Leica Microsystems) at ×10 magnification. Finally, the adipocyte size was quantified using Fiji which is ImageJ software with the add-on Adiposoft plugin (NIH, Bethesda, MD, United States).

### Quantification of Adiponectin, Resistin, and Leptin Concentrations in Mice

Concentrations of the adipokines adiponectin, resistin, and leptin were measured in the serum of WT and *Slc10a6^−/−^* knockout mice in duplicate by ELISA (DuoSet ELISA development systems, R&D Systems, Wiesbaden, Germany). The measurements were repeated for samples exceeding an intra-duplicate variation of 20%.

### Statistical Analysis

All graphs and calculations were prepared using GraphPad Prism software 6.07 (GraphPad Software, La Jolla, CA, United States). Student’s unpaired *t*-test and one-way ANOVA with Tukey’s multiple comparison tests were performed to determine statistical significance. All data were expressed as mean ± standard deviation (SD). *P* < 0.05 was considered statistically significant.

## Results

### Soat Expression in Adipose Tissue

**FIGURE 1:**
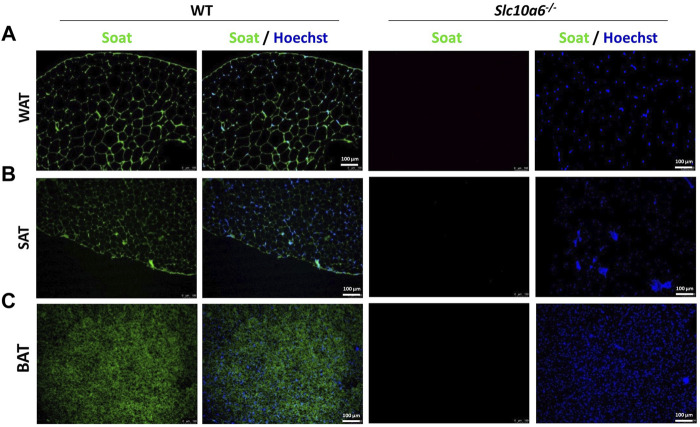
Soat expression in mouse adipose tissues. Immunostaining of the Soat protein in WAT **(A)**, SAT **(B)**, and BAT **(C)** of wild-type and *Slc10a6* ^*−/−*^ knockout mice with the anti-Soat antibody (green). Scale bar: 100 µm. Blue: nuclear counterstaining with Hoechst 33342. WT, wild type; WAT, white adipose tissue; SAT, subcutaneous adipose tissue; BAT, brown adipose tissue.

To explore the role of Soat in intracrine steroid production in peripheral tissues, a *Slc10a6* ^*−/−*^ knockout mouse model was established in a previous study by our group (Bakhaus et al., 2018). In the present study, the expression of Soat protein was investigated in adipose tissues, namely, in white intra-abdominal adipose tissue (WAT), subcutaneous adipose tissue (SAT), and brown adipose tissue (BAT) from WT and *Slc10a6^−/−^* knockout mice. As shown in Figure 1, Soat is abundantly expressed in WAT (Figure 1A), SAT (Figure 1B), and BAT (Figure 1C) in WT mice. As expected, no Soat immunoreactivity was observed in the *Slc10a6^−/−^* knockout mice, which clearly indicated the specificity of the anti-Soat antibody.

### Adipose Tissue Morphology and Serum Adipokine Levels in *Slc10a6* ^*−/−*^ Knockout Mice

**FIGURE 2:**
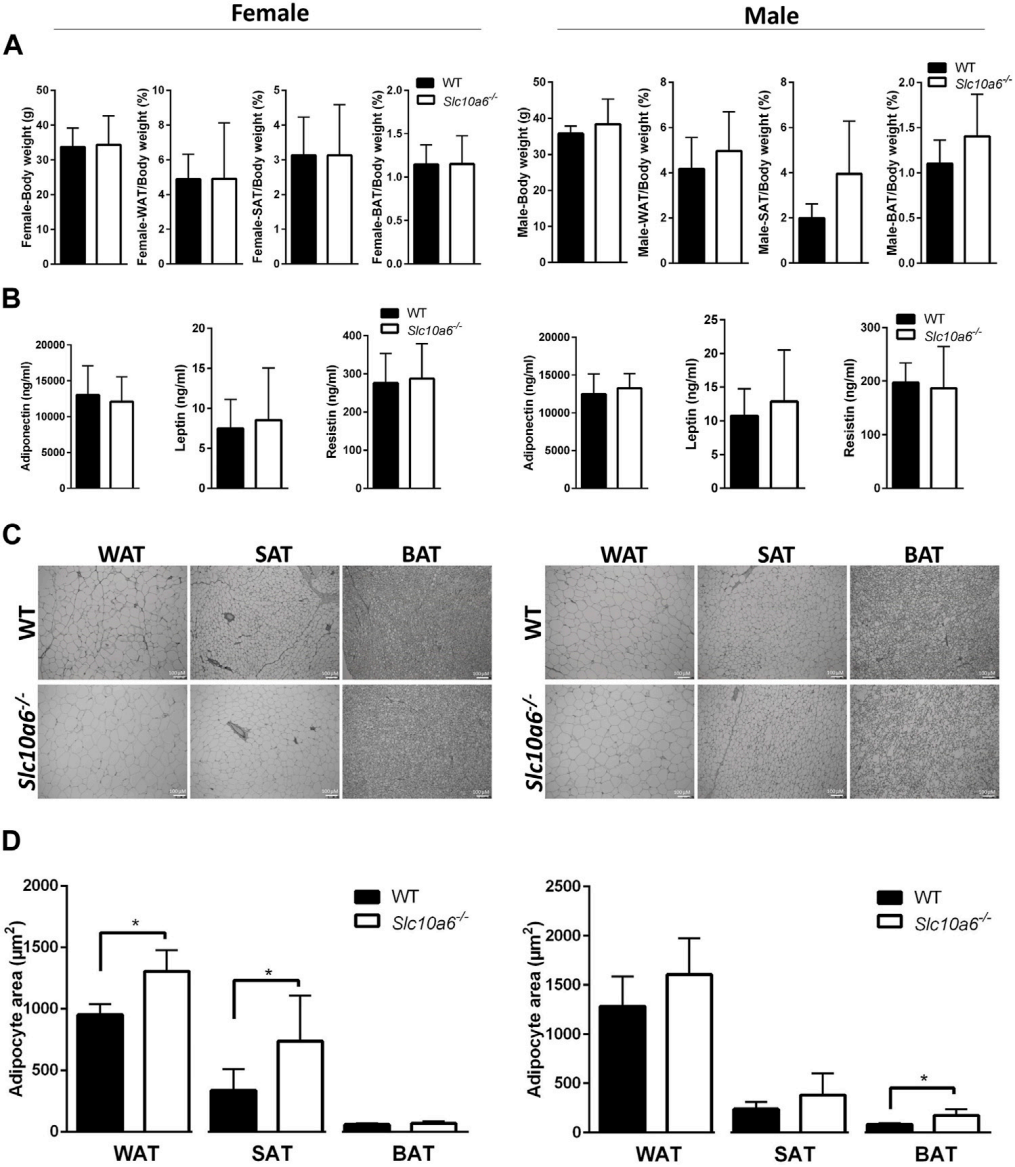
Effects of Soat knockout on body weight, fat tissue weight, and adipocyte size. Adipose tissues of female (left panel) and male (right panel) WT and *Slc10a6* ^*−/−*^ knockout mice were analyzed. **(A)** Weight of adipose tissues (WAT, SAT, and BAT) was normalized for total body weight. Results are shown as means ± SD. **(B)** Serum levels of adiponectin, leptin, and resistin. Data are presented as means ± SD of n = 4 female and n = 6 male mice per genotype. **(C)** Histological presentation and **(D)** quantitative examination of WAT, SAT, and BAT cell morphology. Histological slides after H&E staining were used for quantitative analysis of the mean adipocyte size expressed in µm^2^. Scale bars: 100 μm. Data are presented as mean ± SD. *Significant differences between WT and *Slc10a6* ^*−/−*^ knockout mice indicated by *p* < 0.05 according to Student’s t-test. WT, wild type; WAT, white adipose tissue; SAT, subcutaneous adipose tissue; BAT, brown adipose tissue.

A closer analysis of body weight, adipose tissue weight, and adipocyte size was then carried out in groups of 10 WT (four female and six male) mice and 10 *Slc10a6* ^*−/−*^ knockout (four female and six male) mice. All were derived from het × het breeding and were genotyped as previously reported (Bakhaus et al., 2018). All mice were fed *ad libitum*. As indicated in Figure 2A, all mice showed comparable body weight. WAT, SAT, and BAT specimens were dissected from all mice, and the weights of the adipose tissue were normalized for the total body weight. Although there was a trend for higher WAT, SAT, and BAT ratios in the male *Slc10a6* ^*−/−*^ knockout mice, these differences did not reach the level of significance. In female mice, the WAT, SAT, and BAT ratios were nearly identical between the WT and the *Slc10a6* ^*−/−*^ knockout mice. In addition, serum levels of classical adipokines, namely, adiponectin, leptin, and resistin were analyzed in WT and *Slc10a6* ^*−/−*^ knockout mice by ELISA. As indicated in Figure 2B, there was no significant difference in serum concentrations of adiponectin, leptin, and resistin in female or male mice. In the next step, histological sections after H&E staining (shown in Figure 2C) were used to quantify the mean areas of WAT, SAT, and BAT adipocytes (Figure 2D, data in µm^2^). Interestingly, WAT and SAT in female mice and BAT in male mice showed significantly larger adipocyte size in the *Slc10a6* ^*−/−*^ knockout mice than that in the WT mice.

### LPS Upregulated Soat Expression in 3T3-L1 Adipocytes

**FIGURE 3:**
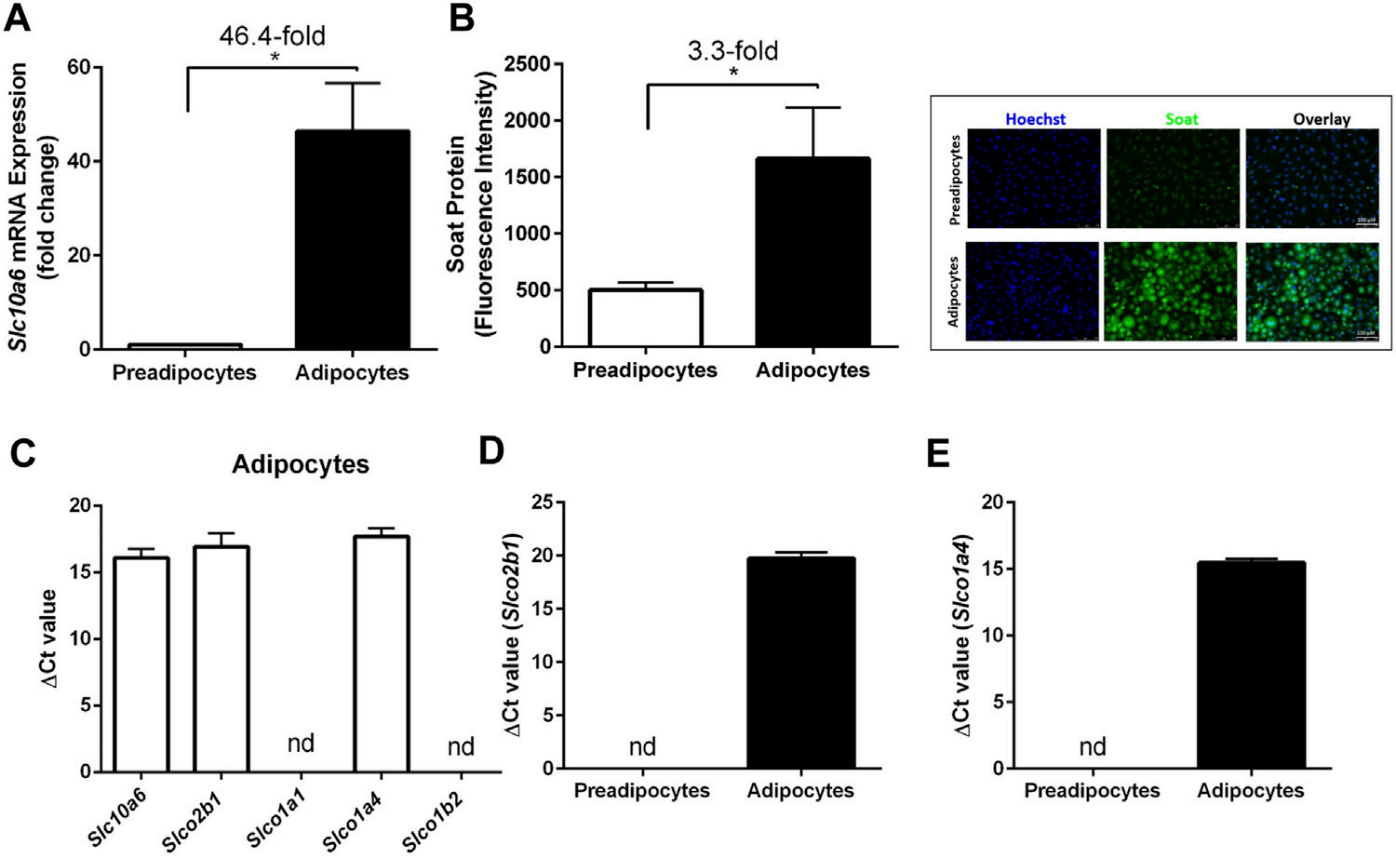
Expression of Soat and other steroid sulfate transporters in 3T3-L1 preadipocytes and adipocytes. **(A)** *Slc10a6* mRNA levels were quantified by qPCR in 3T3-L1 preadipocytes and adipocytes using a *Slc10a6*-specific TaqMan probe. *Gapdh* expression was used to normalize *Slc10a6* expression (ΔCt), and the ΔCT value in preadipocytes was set as the calibrator for 2^−ΔΔCt^ calculation of the fold expression change during adipocyte differentiation. **(B)** Anti-Soat immunofluorescence analysis and quantification of fluorescence intensity in 3T3-L1 preadipocytes and adipocytes. Data in **(B)** represent means ± SD of three independent areas of view (each with 60–74 cells) of three independent replicates. Representative immunofluorescence images of preadipocytes and adipocytes are shown. The cells were fixed and stained with the anti-Soat antibody and the secondary Alexa Fluor 488 goat anti-rabbit antibody (green fluorescence). Hoechst 33342 was used for nuclei staining (blue fluorescence). **(C)** Expression profile of 3T3-L1 adipocytes for different steroid sulfate uptake carriers. **(D)** *Slco2b1* and **(E)** *Slco1a4* mRNA expression in preadipocytes and differentiated adipocytes. Expression data in **(C–E)** are presented as ΔCt values from qPCR analysis and represent carrier expression normalized to *Gapdh* expression. Data are expressed as means ± SD. *Significant differences between preadipocytes and adipocytes indicated by *p* < 0.05 according to Student’s t-test.

Soat expression and lipid accumulation were then analyzed in an adipocyte cell culture model. 3T3-L1 preadipocytes were used and differentiated to adipocytes as described in the *Materials and Methods* section. To examine whether Soat is expressed in 3T3-L1 cells, we first measured *Slc10a6* mRNA levels using qPCR. *Slc10a6* mRNA was detected in 3T3-L1 preadipocytes and showed a significant 46.4-fold upregulation during differentiation into 3T3-L1 adipocytes (Figure 3A). This was confirmed at the protein level by significantly higher immunofluorescence signals with the anti-Soat antibody in mature adipocytes than those in 3T3-L1 preadipocytes (Figure 3B). For comparison, mRNA expression of other steroid sulfate uptake carrier candidates from the Oatp family, namely, *Oatp2b1*, *Oatp1a1*, *Oatp1a4,* and *Oatp1b2* was analyzed in 3T3-L1 adipocytes using qPCR. As shown in Figure 3C, only the expression of the *Slco2b1* and *Slco1a4* mRNAs was detected, while the *Slco1a1* and *Slco1b2* mRNAs were not expressed in the 3T3-L1 adipocytes. Unlike *Slc10a6*, *Slco2b1* (Figure 3D) and *Slco1a4* (Figure 3E) mRNAs were not detected in 3T3-L1 preadipocytes.

**FIGURE 4:**
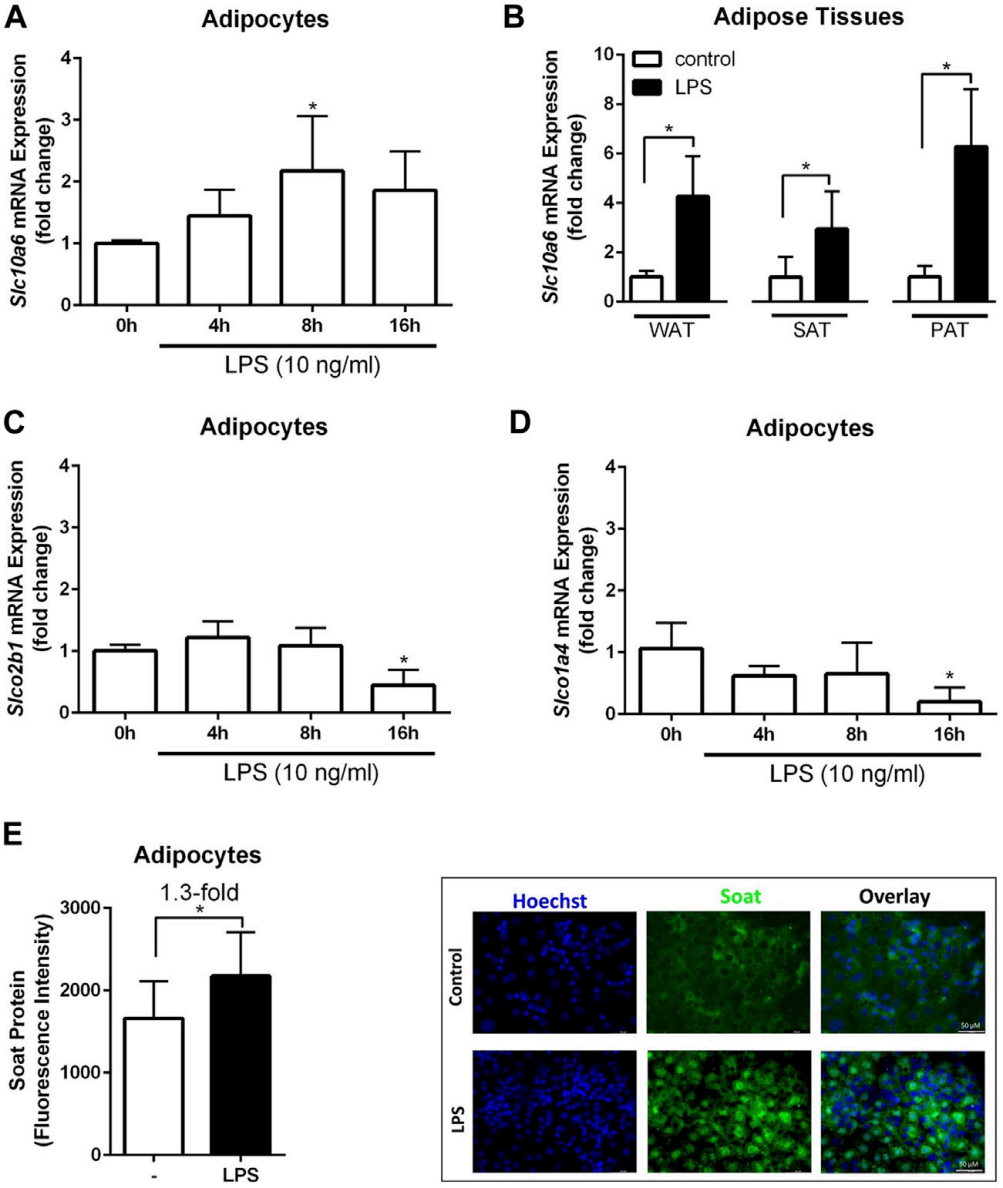
Expression analysis of steroid sulfate uptake carriers in adipocytes and adipose tissue in response to LPS. The mRNA expression levels of *Slc10a6* **(A)**, *Slco2b1* **(C)**, and *Slco1a4* **(D)** were quantified using qPCR in LPS-induced 3T3-L1 adipocytes. 3T3-L1 adipocytes were treated with 10 ng/ml LPS for the indicated time periods. Data represent means ± SD of triplicate determination of representative experiments **(B)** *Slc10a6* mRNA expression in LPS-treated mice for WAT, SAT, and PAT (n = 6). Data represent means ± SD. All carrier mRNA expression levels were normalized to *Gapdh* expression. Soat protein expression in **(E)** represents means ± SD of three independent areas of view (each with 60–74 cells) of three independent replicates. The mature adipocytes were treated with 10 ng/ml LPS for 24 h. Representative immunofluorescence images are shown. Cells were fixed and stained with the anti-Soat antibody and the secondary Alexa Fluor 488 goat anti-rabbit antibody (green fluorescence). Hoechst 33342 was used for nuclei staining (blue fluorescence). *Significant differences indicated by *p* < 0.05 using one-way ANOVA **(A**, **C** and **D)** or Student’s t-test **(B** and **E)**. LPS, lipopolysaccharide; WAT, white intra-abdominal adipose tissue; SAT, subcutaneous adipose tissue; PAT, perirenal adipose tissue.

A previous study showed that Slc10a6 mRNA levels were significantly induced by LPS in the mouse liver and WAT (Kosters et al., 2016). To further investigate the response of peripheral intracrine tissues to inflammation, gene expression analysis of steroid sulfate carriers was performed in LPS-induced 3T3-L1 adipocytes and mouse adipose tissues. As shown in Figure 4, LPS treatment of 3T3-L1 adipocytes significantly increased *Slc10a6* mRNA (Figure 4A) and Soat protein expressions (Figure 4E). In contrast, LPS treatment of the 3T3-L1 adipocytes significantly induced downregulation of the *Slco2b1* (Figure 4C) and *Slco1a4* (Figure 4D) mRNA expression at least at 16 h, indicating that LPS-induced upregulation was specific for Soat. In addition, *Slc10a6* mRNA expression was analyzed in WAT, SAT, and PAT specimens of LPS-treated WT mice and in untreated control mice. LPS was injected intraperitoneally at 1 µg LPS per animal. As shown in Figure 4B, *Slc10a6* mRNA expression was significantly induced by LPS treatment in all types of adipose tissue, namely, WAT, SAT, and PAT.

### DHEAS-Induced Lipolysis in LPS-Treated 3T3-L1 Adipocytes

**FIGURE 5:**
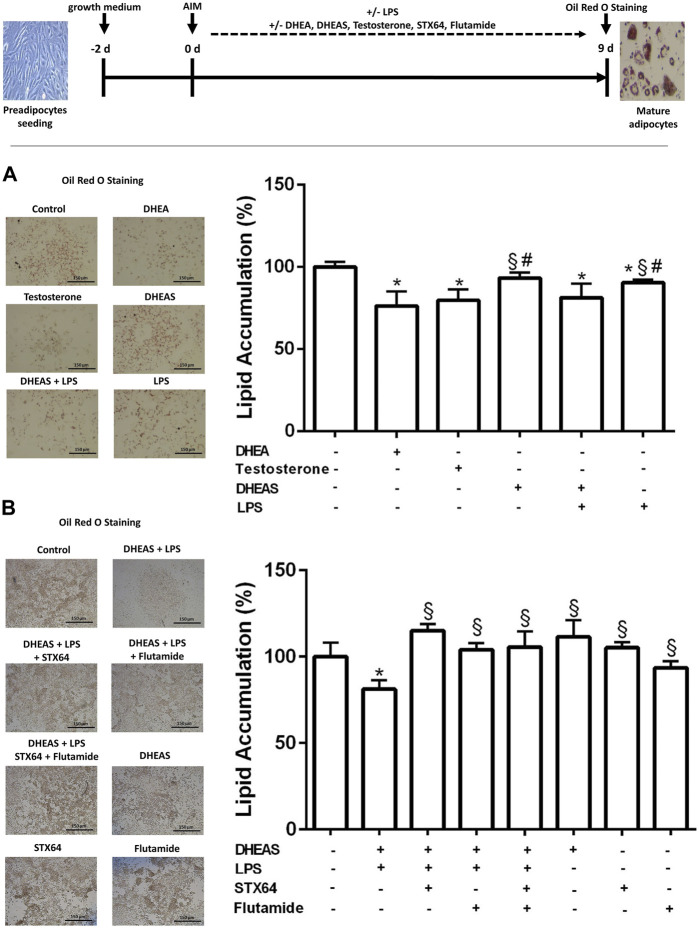
Effect of DHEAS on lipid accumulation in LPS-induced 3T3-L1 adipocytes. Scheme of the experimental procedure for 3T3-L1 differentiation from preadipocytes to mature adipocytes over 9 days and cell treatment. Cell differentiation was induced by hormonal supplementation (AIM) as described in the *Materials and Methods* section. 3T3-L1 preadipocytes were incubated with DHEA (1 µM), testosterone (100 nM), DHEAS (10 µM), STX64 (10 µM), or flutamide (1 µM) in the presence or absence of LPS for the entire 9-day differentiation period, and every 2 days the medium was changed with fresh AIM containing the respective compounds. On day 9, the cells were stained with Oil Red O. Representative photomicrographs of Oil Red O staining in mature 3T3-L1 adipocytes are shown (scale bar: 150 μm). Lipid accumulation was measured by Oil Red O extraction and quantification using a microplate reader. Data are expressed as mean ± SD of three replicates from **(A)** three independent experiments and from **(B)** one representative experiment. Significant differences are indicated by *p* < 0.05 using one-way ANOVA with Tukey’s multiple comparisons test: *significantly different compared to control; ^§^significantly different compared to LPS + DHEAS; ^#^significantly different compared to DHEA and testosterone. AIM, adipogenesis-inducing media.

To investigate the role of Soat in modulating DHEAS-mediated effects on adipogenesis, 3T3-L1 cells were treated with LPS through the entire differentiation process of 9 days. Furthermore, DHEA, DHEAS, testosterone, STX64, and flutamide were incubated as indicated in the experimental procedure scheme (Figure 5). Lipid accumulation was measured by extraction and quantification of Oil Red O dye after completion of 3T3-L1 differentiation on day 9. Representative images of Oil Red O dye staining are presented in Figure 5A,B. Treatment with DHEA and testosterone significantly reduced the accumulation of intracellular lipid droplets. Treatment with DHEAS alone also reduced the lipid accumulation but without reaching statistical significance. However, DHEAS also significantly reduced lipid accumulation after co-incubation with LPS, and this effect was significantly stronger than that with LPS treatment alone. These results suggest that DHEAS negatively regulates lipid accumulation in differentiated adipocytes through LPS-induced Soat expression. The DHEAS-mediated inhibition of lipid accumulation observed in the LPS-exposed 3T3-L1 cells was reversed in the presence of STX64, a specific inhibitor of STS (Figure 5B), suggesting that conversion of DHEAS to DHEA by STS is essential for this process. In addition, the androgen receptor (AR) antagonist flutamide reversed this effect (Figure 5B), suggesting that an AR-dependent pathway is involved.

## Discussion

In the present study, we characterized for the first time the potential role of the SOAT/Soat steroid sulfate uptake carrier in adipose tissue and adipocytes. Transporters such as Soat are responsible for the cellular uptake of negatively charged sulfated steroids and, therefore, can be regarded as gatekeepers in the plasma membrane for the intracellular functions of these precursor molecules. In principle, there are several candidates for steroid sulfate uptake carriers from the OATP/Oatp and OAT/Oat carrier families that show widespread tissue expression (Roth et al., 2012). Furthermore, the hepatic bile acid carrier Na^+^/taurocholate co-transporting polypeptide (NTCP/Ntcp) is involved in the uptake of sulfated steroids by hepatocytes (Geyer et al., 2006). The Soat carrier is unique among these carriers for several reasons: (I) In contrast to OATPs/Oatps and OATs/Oats, SOAT/Soat mediates a sodium-coupled active transport of its substrates into cells; (II) this carrier is very substrate-specific and exclusively transports sulfated steroid hormones, whereas the other carriers also have bile acids and drugs among their substrates and, thus, are multispecific; (III) SOAT/Soat is highly expressed in many peripheral tissues known to be steroid responsive, and in some of these tissues SOAT/Soat is even coexpressed with STS (e.g. in the placenta) (Geyer et al., 2007; Fietz et al., 2013; Grosser et al., 2013; Schweigmann et al., 2014; Bakhaus et al., 2018). All these points make SOAT/Soat an interesting candidate for intracrine steroid regulation in peripheral tissues and organs, such as adipose tissue.

SOAT/Soat transports all 3′ and 17′ monosulfated steroids that are physiologically relevant and of these, in humans, DHEAS is quantitatively the most relevant and dominant precursor (Orentreich et al., 1984) that is used to synthesize androgens and estrogens, such as testosterone, dihydrotestosterone, estrone, and 17β-estradiol, *via* STS and other steroidogenic enzymes (Khalil et al., 1993; Labrie et al., 1995; Reed et al., 2005). STS is expressed in preadipocytes and mature adipocytes (Marwah et al., 2006; McNelis et al., 2013) and, thus, could exert intracrine effects on adipogenesis in combination with Soat, which is highly expressed in WAT, SAT, and BAT adipose tissue, at least in mice (Figure 1).

**FIGURE 6:**
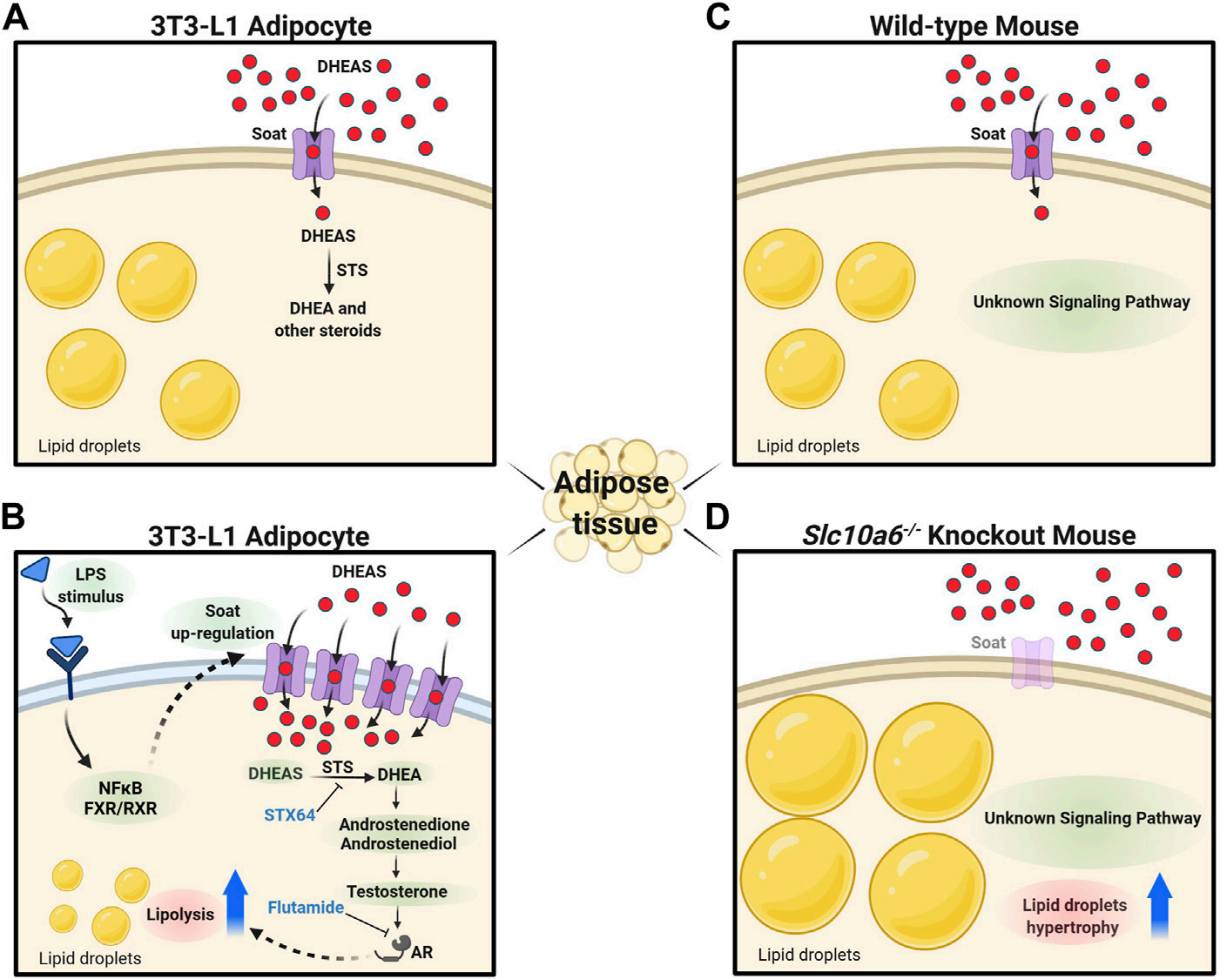
Proposed role of Soat in lipid droplet formation and steroid-triggered lipolysis. **(A)** Soat expression in the plasma membrane of 3T3-L1 adipocytes and uptake of DHEAS under physiological conditions. **(B)** Upregulation of Soat expression by LPS treatment through JNK and NF-κB–dependent pathways increases DHEAS uptake and stimulation of lipolysis *via* an AR-dependent pathway. The lipolytic effect of DHEAS could be blocked by STX64 and flutamide. **(C)** As DHEAS levels are low in mice, Soat may mediate uptake of other relevant sulfated steroids here. **(D)** Absence of steroid sulfate uptake through Soat in *Slc10a6* ^*−/−*^ knockout mice could lead to the hypertrophy of lipid droplets in adipocytes *via* hitherto unknown signaling pathways. DHEAS, dehydroepiandrosterone sulfate; DHEA, dehydroepiandrosterone; STS, steroid sulfatase; STX64, STS inhibitor; LPS, lipopolysaccharide; NF-κB, nuclear factor “kappa-light-chain-enhancer” of activated B-cells; FXR, farnesoid X receptor; RXR, retinoid X receptor; AR, androgen receptor; flutamide, androgen receptor antagonist. The figure was created with biorender.com.

Considering our current knowledge about SOAT/Soat, we assume a functional role in the local supply of adipose tissue with sulfated steroids, which could then be involved in the regulation of various physiopathological processes such as obesity (Figure 6). There are numerous studies showing that serum DHEAS levels are negatively correlated with obesity (Al-Harithy, 2003; Hernández-Morante et al., 2008; Gómez-Santos et al., 2012; Jaff et al., 2015; Sayın et al., 2020). However, the role of SOAT in this process is difficult to demonstrate in human patients. Thus, effects of DHEA/DHEAS on adipocytes are often analyzed in the mouse 3T3-L1 cell line to investigate adipogenesis, lipogenesis, and lipolysis (Green and Meuth, 1974; Green and Kehinde, 1975), considering that rodents have much lower circulating concentrations of these hormones (Guillemette et al., 1996). In addition, much of our knowledge concerning preadipocyte biology and adipocyte differentiation has been obtained using 3T3-L1 cells. In the present study, we also used WT and *Slc10a6* ^*−/−*^ knockout mice to investigate the role of Soat on body weight, fat tissue weight, adipocyte size, and serum adipokine levels *in vivo* in male and female mice. Our results demonstrate that the knockout of Soat expression did not influence total body weight and the weight of adipose tissues in mice fed *ad libitum*. These results are consistent with our previous findings in *Slc10a6* ^*−/−*^ knockout mice (Bakhaus et al., 2018). We also did not find any significant differences in the gain or loss of fat-free mass with weight changes and the serum levels of the adipokines leptin, adiponectin, and resistin (Figure 2). These adipokines originate primarily from fat deposits and exert essential regulatory functions in adipose tissue, especially under different metabolic conditions such as obesity, fasting, and diabetes (Galic et al., 2010; Coelho et al., 2013; Leal and Mafra, 2013). Adiponectin is the most abundant circulating adipokine (Yamauchi et al., 2001). In some studies, the serum levels of adiponectin are inversely correlated with those of adipose tissue. Arita et al. (1999) reported that plasma adiponectin concentrations decrease with obesity in humans. Similarly, male patients with morbid obesity present higher serum levels of DHEAS than obese women, while women exhibit higher plasma adiponectin values than male patients (Hernández-Morante et al., 2006). This study by Hernández-Morante et al. (2006) also demonstrated the significant effect of DHEAS on the upregulation of adiponectin in visceral adipose tissue. Furthermore, DHEAS replacement therapy in postmenopausal women resulted in decreased plasma levels of adiponectin (Gómez-Santos et al., 2012). Conversely, reduction in leptin and an upregulation of resistin gene expression have been described in adipose tissue after DHEA administration (Kochan and Karbowska, 2004). One study reported that rats treated with DHEA express elevated serum concentrations of resistin and DHEAS but decreased serum levels of leptin and adiponectin (Pérez-de-Heredia et al., 2008). It is noteworthy that the lack of Soat *per se* had no effect on serum adiponectin, leptin, and resistin in our study. However, it must be considered that these mice were fed a standard diet *ad libitum*. Future experiments including high-fat diet feeding of *Slc10a6* ^*−/−*^ knockout mice and additional DHEAS treatment could be helpful in fully elucidating the role of Soat in adipogenesis.

It is noteworthy that *Slc10a6* ^*−/−*^ knockout mice were characterized by a significant increase in adipocyte size in WAT and SAT of female and in BAT of male mice compared to WT control animals (Figure 2D). The large interindividual variability observed in adipocyte size suggests that the propensity for fat cell hypertrophy in each fat compartment may differ between the groups studied. Interestingly, male *Slc10a6* ^*−/−*^ knockout mice showed significantly higher serum cholesterol sulfate levels than their wild type controls, with no significant difference found for female mice (Bakhaus et al., 2018). Approximately, 25% of total cholesterol is stored in adipose deposits in normal weight humans, while this rate can rise to 50% in obese individuals (Prattes et al., 2000). Lipid droplet expansion requires substantial amounts of cholesterol, triggering the transfer of caveolin proteins from the plasma membrane to lipid droplets to facilitate their enlargement (Chui et al., 2005; Robenek et al., 2011). Furthermore, Dalla Valle et al. (2006) demonstrated STS expression at the levels of mRNA, protein, and catalytic activity in human adipose tissue, whereas sulfotransferase expression was not found. Thus, higher serum cholesterol sulfate levels in males may have masked differences in adipocyte size. In this context, further studies are needed including analytical steroid profiling of serum and adipose tissue from WT and *Slc10a6* ^*−/−*^ knockout mice.

The expression of *Slc10a6* mRNA and Soat protein was upregulated in 3T3-L1 cells after differentiation from preadipocytes into mature adipocytes (Figure 3). In addition, the mRNA expression of other potential steroid sulfate uptake carriers in adipocytes was analyzed, and *Slco2b1* and *Slco1a4* were also found to be expressed at low levels, while *Slco1a1* and *Slco1b2* mRNAs are not detectable in mature adipocytes. Dalla Valle et al. (2006) and McNelis et al. (2013) reported that mRNA expression of the human OATP carriers OATP-B (*SLCO2B1*), OATP-D (*SLCO3A1*), OATP-E (*SLCO4A1*), and of STS, could be detected in human adipose tissue and in cultured preadipocytes and adipocytes, whereas OATP-A (*SLCO1A2*), OATP-C (*SLCO1B1*), OATP-8 (*SLCO1C3*), OAT3 (*SLC22A8*), and OAT4 (*SLC22A11*) were not detected. In addition, Dalla Valle et al. (2006) analyzed the expression of steroidogenic enzymes and related proteins such as cytochromes P450scc, P450c17, P450arom, steroidogenic factor 1 (SF-1), and steroidogenic acute regulatory protein (StAR), which are involved in the *de novo* biosynthesis of steroid hormones from cholesterol. They showed that none of these enzymes could be detected at the mRNA level, while STS mRNA was expressed in samples from cultured preadipocytes, adipocytes, and adipose tissues. The absence of these essential enzymes required for cholesterol-mediated estrogen and androgen biosynthesis suggests that uptake carriers for sulfated steroid precursors, indeed, might be essential for the regulation of the adipose tissue by steroids.

A global gene expression analysis in mice demonstrated that Soat is among the most strongly induced genes by LPS in the liver and macrophages (Kosters et al., 2016). In addition, *Slc10a6* mRNA expression was upregulated in the liver of mice treated with the cytokine IL-1β. The inflammation-mediated upregulation of Soat expression was likely modulated by the nuclear receptor farnesoid X receptor (FXR), the retinoid X receptor (RXR), and glucocorticoid receptor (GR), which were expressed by all tissues, including white and brown adipose tissues. Since the testes have higher Soat expression than other tissues, the testes were used in this study as the control tissue. However, Soat shows higher expression in the BAT and is less expressed in WAT of untreated mice when compared to that in the testes. In contrast, *Slc10a6* mRNA levels are upregulated in WAT and downregulated in BAT specimens of LPS-induced mice (Kosters et al., 2016). We found that LPS-elicited inflammation induced a significant increase in *Slc10a6* mRNA expression in WAT, SAT, and PAT specimens of mice *in vivo* and in mature 3T3-L1 adipocytes *in vitro* (Figure 4). In contrast, the mRNA transcripts for *Slco2b1* and *Slco1a4* were downregulated in LPS-treated mature 3T3-L1 adipocytes.

DHEA treatment suppresses both proliferation and adipocyte differentiation of 3T3-L1 cells and inhibits AIM-induced lipid accumulation in 3T3-L1 adipocytes (Lea-Currie et al., 1998; Fujioka et al., 2012; Yokokawa et al., 2020). This finding was confirmed in the present study. Marwah et al. (2006) showed that DHEA is metabolized into adiol and testosterone in 3T3-L1 adipocytes. Furthermore, Singh et al. (2006) demonstrated that testosterone and dihydrotestosterone, in a dose-dependent manner, caused inhibition of lipid accumulation in 3T3-L1 differentiated for 12 days. Moreover, this inhibitory effect was significantly blocked by the AR antagonist flutamide (Singh et al., 2006). These studies suggest that testosterone and dihydrotestosterone both play a role in adipogenic differentiation of 3T3-L1 cells *via* the AR. Lea-Currie et al. (1998) showed that DHEAS, in contrast to DHEA, had no impact on preadipocyte proliferation and differentiation. These studies corroborate our findings in adipocytes, demonstrating that DHEA, but not DHEAS, suppresses both proliferation and lipid accumulation. The lack of effects of DHEAS on murine 3T3-L1 preadipocytes and adipocytes may be due to the low expression levels of steroid sulfate uptake transporters. In this context, another interesting observation of our study was the LPS-induced upregulation of Soat in adipocytes and the decrease in adipocyte differentiation and AIM-induced lipid accumulation by DHEAS, accompanied by LPS-mediated upregulation of Soat in 3T3-L1 cells (Figure 5). Although lipid accumulation was also significantly reduced by LPS treatment alone, we observed a significantly stronger effect of LPS treatment combined with DHEAS. Moreover, we tested whether DHEAS-mediated inhibition of lipid accumulation was related to downstream steroid metabolites. We observed that both STS inhibition by STX64 and AR inhibition by flutamide completely abolished the DHEAS-mediated effects, clearly indicating that conversion of DHEAS to DHEA and subsequent androgens is involved in this process (Figure 5B). It is well known that obesity due to excess fat accumulation is associated with varying degrees of chronic inflammation in adipose tissue. This leads to hypoxia, adipocyte cell death, and modified secretion of adipokines (Sanada et al., 2016) and finally ends in chronic morbidities characterized by metabolic inflammation (Handschin and Spiegelman, 2008; Makki et al., 2013). The results of the present study suggest that the inflammation-mediated overexpression of Soat and subsequently increased uptake of DHEAS finally lead to inhibition of adipocyte differentiation and reduced lipid accumulation *via* the AR, as depicted in Figure 6.

In conclusion, Soat is a specific uptake carrier for sulfated steroids with significant expression levels in adipose tissues and a proposed role in obesity. To clarify this role *in vivo*, *Slc10a6* ^*−/−*^ knockout mice were analyzed. We observed that LPS-induced inflammation increased the expression of Soat in adipocytes and adipose tissues, contributing to lipolysis by increased uptake of the Soat substrate DHEAS, while deletion of Soat caused adipocyte hyperplasia in mice. We are aware that our study has some limitations. The most important is that the steroid profiles in the adipocytes could not be analyzed, so it was not possible to determine how DHEAS was exactly converted to active androgens.

## Data Availability

The original contributions presented in the study are included in the article/Supplementary Material, further inquiries can be directed to the corresponding author.
